# Clinical report: variable phenotypic expression in a large sibling cohort with a deletion of 4p16.1

**DOI:** 10.1002/ccr3.638

**Published:** 2016-08-18

**Authors:** Carrie Guy, Xianfu Wang, Xianglan Lu, Jin Lu, Shibo Li

**Affiliations:** ^1^ University of Oklahoma Health Sciences CenterOklahoma CityOklahomaUSA

**Keywords:** 4p16.1, chromosome 4, deletion, microarray, *WFS1*

## Abstract

We report a half‐sibling cohort with deletion of 4p16.1, astigmatism, gross and fine motor delay, variable intellectual disability, and variable behavioral concerns. However, two siblings without the deletion also had learning delays and psychological concerns. Thus, variable phenotypic expression was seen and the significance of deletion of 4p16.1 remains unclear.

## Introduction

We present a large, maternal half‐sibling cohort with a familial deletion of 4p16.1 with variable phenotypic presentation. To date, this is the first large, half‐sibling cohort with a deletion of 4p16.1 to be reported. Deletions of 4p have been previously reported and with increased sensitivity of advancing microarray technology, more specific genotype–phenotype associations have been described. With this report, we aim to contribute to the understanding of the clinical significance of deletions in the 4p region.

The deleted 4p16.1 region in this sibling cohort includes the last exon of *JALMP1* and the entire gene region of *LOC28548*,* WFS1*, and *PPP2R2C*. The *WFS1* gene (OMIM #606201) codes for the protein wolframin, and is associated with Wolfram syndrome (OMIM #222300). Wolfram syndrome is an autosomal recessive disorder characterized by variable features, including diabetes insipidus, diabetes mellitus, optic atrophy, and deafness 1 . Mutations in *WFS1* are also associated with autosomal dominant optic atrophy with hearing impairment 2 and in autosomal dominant low‐frequency sensorineural hearing impairment (LFSNHI) 3, 4. Mutations in *WFS1* are also found in patients with diabetes mellitus and hearing impairment 5, in a single family with autosomal dominant optic neuropathy, deafness, and no glucose intolerance 6, and in eight families with autosomal dominant isolated optic atrophy and hearing loss 7.

Autosomal dominant Wolfram‐like syndrome (OMIM #614296) is also associated with mutations in *WFS1*. Wolfram‐like syndrome is characterized by congenital progressive hearing impairment, diabetes mellitus, and optic atrophy 8. Previous clinical reports of patients with Wolfram‐like syndrome have included several reported families with novel missense mutations in *WFS1* and progressive hearing loss, while frequency of reports of optic atrophy and impaired glucose regulation vary 2, 5, 6. The association of Wolfram‐like syndrome and psychiatric diagnoses, including bipolar disorder, schizophrenia, and depression is a point of discussion 1, 7. Review of reported cases of Wolfram‐like syndrome revealed that 55% of all mutations reported are null alleles, suggesting pathogenicity due to a deficiency in wolframin. Over 90 different mutations have been reported for this gene 1.

Neither *JALMP1* nor *LOC28548* are annotated in OMIM (http://www.ncbi.nlm.nih.gov/omim, accessed 11/4/2015) and a search on PubMed (http://www.ncbi.nlm.nih.gov/pubmed, accessed 11/4/2015) revealed no published clinical reports, searching by either gene name as keyword. One prior report described a family with six members with mild intellectual disability and who carried a reciprocal translocation, t(4;6)(p16.1;q22), that interrupted the *PPP2R2C* gene (OMIM 605997) and the *LAMA2* gene (OMIM #156225) 9.

The clinical significance of deletion of 4p16.1 remains unclear, as there are few reports of this interstitial deletion on the short‐arm of chromosome 4. No prior deletions of a similar size were identified in DECIPHER (DECIPHER, https://decipher.sanger.ac.uk/), ClinVar (www.ncbi.nlm.nih.gov/clinvar/) (accessed 6/5/2015), or Database of Genomic Variants (http://dgv.tcag.ca/dgv/app/home, accessed 11/14/15). Thus, to the best of our knowledge, this is the first clinical report of deletion of 4p16.1 in a large, sibling cohort. To contribute to the growing understanding of cytogenetic deletions, we present a report of a large, maternal half‐sibling cohort with deletion of 4p16.1.

## Participants and Methods

### Participants

**Figure 1:**
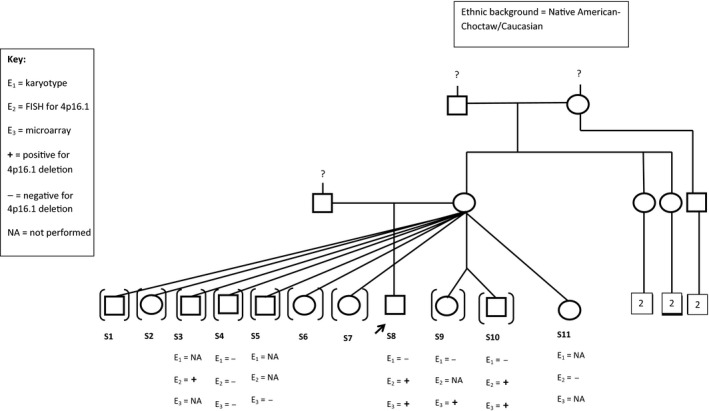
Pedigree of half‐sibling cohort.

A large sibling cohort of eleven maternal half‐siblings was identified in the University of Oklahoma Health Sciences Center (OUHSC) Clinical Molecular and Cytogenetics Laboratory database after identifying a deletion of 4p16.1 in three of the half‐siblings (Fig. 1). Sibling‐8 was the first sibling to come to attention in the OUHSC Clinical Genetics Laboratory. Microarray analysis performed (Agilent 2x400K V.1.0, Agilent Technologies Inc.), revealed a 405Kb deletion on chromosome 4p16.1 (6,175,320–6,580,382). Simultaneously, microarray analysis results for Sibling‐9 and Sibling‐10 also revealed the same 405Kb deletion on chromosome 4p16.1 (6,175,320–6,580,382). Due to the uncommon deletion identified and similar surnames, ordering providers for the patients were contacted and it was confirmed that they were from the same large, half‐sibling cohort. Fluorescence in situ hybridization (FISH) analysis was performed (using BAC clone probe RP11‐29N16) on the sample from Sibling‐8 for future testing of additional siblings and was found to be informative for the familial deletion. Through communication with the ordering pediatricians as well as the foster and adoptive parents, the siblings were seen in the OUHSC Pediatric Genetics Clinic for genetic counseling regarding the family history of 4p16.1 deletion. Pre‐ and post‐test genetic counseling was conducted and genetic testing, as applicable, was ordered. Microarray analysis or FISH analysis for 4p16.1 was performed. The pediatricians for three of the participants ordered genetic testing independent of the OUHSC Pediatric Genetics Clinic. A summary of the genetic tests performed on each participant is found on Table 1. Adoptive guardians were contacted and written informed consent was obtained for seven of the siblings to enter the Chromosomal Anomalies Registry (IRB #2250). Two of the adoptive families, each with two of the half‐siblings (Sibling‐1 and Sibling‐2 in one family and Sibling‐6 and Sibling‐7 in the other), declined participation in the Chromosomal Anomalies Registry or were lost to follow‐up and therefore are not described here.

**Table 1:** Comparison of clinical findings of siblings with and without the familial deletion

Clinical Feature	S‐3	S‐4	S‐5	S‐8	S‐9	S‐10	S‐11	Mother	Wolfram‐like syndrome
4p16.1 deletion	+FISH^a^	−FISH^b^ −aCGH^c^ 46, XY	−aCGH	+aCGH^d^ +FISH 46, XY	+aCGH 46, XY	+aCGH +FISH 46, XX	−FISH	?^e^	
Progressive healing loss	Normal screen	Normal screen		Normal screen	Inconclusive	Inconclusive			+^f^
Diabetes mellitus								+	+
Ocular conditions	Normal exam	Glasses‐diagnosis unknown		Hypermetropia	Astigmatism	Astigmatism		Blind in left eye	Optic atrophy
Psychiatric or behavioral concerns	Anxiety? Behavior ADHD Sensory processing	Behavior Sensory processing	Behavior ADHD	Behavior	Behavior	Anxiety? Behavior Sensory processing		+	+
Intellectual disability	Dyslexia Speech delay Auditory processing disorder	Developmental delay Mixed receptive‐ expressive language disorder Auditory processing disorder	Speech delay Learning delay	Speech delay	Learning delay	Learning delay		+	
Asthma/allergies	Asthma Allergies	Asthma			Milk allergy?	Food allergy?			
Neurological		Gross/fine motor delay		Gross/fine motor delay	Gross/fine motor delay Involuntary movement	Gross/fine motor delay Low tone			

a Deletion detected by Fluorescence in situ hybridization (FISH) analysis.

b Deletion not detected by Fluorescence in situ hybridization (FISH) analysis.

c Deletion not detected by microarray analysis.

d Deletion detected by microarray analysis.

e Clinical feature not confirmed.

f Clinical feature reported present.

Sibling‐3 is a 10‐year‐old male diagnosed with disruptive behavior disorder, inattention, impulsive oppositional disorder, attention deficit hyperactivity disorder (ADHD), and dyslexia. He is reported to exhibit signs of anxiety, though a formal diagnosis has not been made. He currently attends public school in the 4th grade and receives reading and math tutoring and speech therapy. He has been diagnosed with auditory processing disorder and has normal hearing and vision evaluations. He has been diagnosed with asthma and the following allergies: latex, peanuts, wheat, eggs, tree nuts, amoxicillin, dogs, cats, grass, kiwi, green vegetables, watermelon, bananas, apples, and seafood. He has a history of febrile seizures with the last one occurring at 6 years of age. His adoptive parent reported normal growth and normal fine and gross motor skill development. He is reported to be a “picky eater” with aversion to certain textures. FISH analysis for 4p16.1 was consistent with the familial deletion.

Sibling‐4 is an 8‐year‐old male diagnosed with developmental delay, sensory processing disorder, speech delay, mixed receptive‐expressive language disorder, auditory processing disorder, fine and gross motor delay, and possible fetal alcohol spectrum disorder. He has notable behavioral concerns, including frustration and anger, and is sensitive to food textures. He has a diagnosis of asthma and has worn glasses (diagnosis unknown) since he was 3 years of age. Routine karyotype analysis and microarray CGH (NimbleGen 385K chip, genome assembly Mar 2006 [NCBI 36/Hg18]) performed in 2009 were both normal. FISH analysis for the familial 4p16.1 deletion was negative.

Sibling‐5 is a 7‐year‐old male with unknown prenatal history. He has speech and learning delays and has been diagnosed with ADHD. No fine or gross motor delays were reported. His behavior, as reported by his adoptive parent, is to be “babying” and “defiant.” Microarray analysis results (Agilent 2x400K V.1.0, Agilent Technologies Inc.) did not reveal the family deletion nor any other significant (genomic copy number losses of >200 kb and gains of >500 kb outside a known clinically significant region, that include at least one OMIM annotated gene) gains or losses.

Sibling‐8 is a 2‐year‐old male with unknown prenatal history and reports of exposure to unspecified seizure medication, cigarette use, and possible alcohol and drug use. He passed the newborn hearing screening test. He was removed from his biological mother's care at 13 months and was in foster care until 22 months of age, before returning to the care of his biological father. Early concerns for speech, fine motor, gross motor, and behavioral concerns included aggression and self‐harm behaviors (including biting, head banging, and pinching). Evaluation by developmental pediatrician at 16 months revealed, he did not meet the criteria for Fetal Alcohol Syndrome. Bilateral epicanthal folds, normal head circumference, and normal cognitive testing were reported; however, he qualifies for and receives speech therapy. His ophthalmology evaluation revealed hypermetropia, and he has short stature, flat, wide nasal bridge with mild hooding of the eyelids. Microarray analysis performed (Agilent 2x400K V.1.0, Agilent Technologies Inc.), revealed a 405Kb deletion on chromosome 4p16.1 (6,175,320–6,580,382). Fluorescence in situ hybridization (FISH) analysis was performed (using BAC clone probe RP11‐29N16) and was found to be informative for the familial deletion. Routine karyotype analysis revealed a normal male (46, XY) karyotype.

Sibling‐9 is a 1‐year‐old male twin with unknown prenatal history. He had early delay of milestones, which decreased with physical and occupational therapy, and still continues to have gross and fine motor delay. No behavioral concerns were reported by the adoptive parent, but he is reported to not “self‐soothe well” and to have difficulty sleeping. There is a possibility of hearing loss, though testing has been inconclusive. His vision exam revealed astigmatism and he is reported to have a possible milk allergy. Microarray analysis performed (Agilent 2x400K V.1.0, Agilent Technologies Inc.), revealed a 405Kb deletion on chromosome 4p16.1 (6,175,320–6,580,382) and routine karyotype analysis revealed a normal male (46, XY) karyotype.

Sibling‐10 is a 1‐year‐old female twin with unknown prenatal history. She had early delay of milestones, which was reported to have decreased with physical and occupational therapy, and she continues to have gross and fine motor delay. Her adoptive parents have not reported any behavioral concerns, but she is reported to have “sensory issues,” refusing to touch certain items and shows signs of anxiety. She receives speech therapy for feeding difficulty. She is reported to have an allergic rash reaction to strawberries. She receives physical therapy for low tone and occupational therapy for motor delay. Hearing evaluation revealed “shallow movements in one ear” and reevaluation is pending. Her vision exam revealed astigmatism. Microarray analysis performed (Agilent 2x400K V.1.0, Agilent Technologies Inc.), revealed a 405Kb deletion on chromosome 4p16.1 (6,175,320–6,580,382) and FISH analysis revealed the familial 4p16.1 deletion. Routine karyotype analysis revealed a normal female (46, XX) karyotype.

Sibling‐11 is a 5‐week‐old female, with unknown prenatal care with reports of tobacco use. An abnormal echocardiogram revealed pulmonary arterial stenosis. She is reported to be “very stiff” and is pending a physical therapy evaluation. FISH analysis was normal for the familial 4p16.1 deletion.

The maternal family history of these siblings is largely unknown, but there is a possible history of bipolar disorder. The mother's medical history is unknown, but reported by the adoptive parents to include history of seizures, asthma, diabetes, blindness of unknown etiology in the left eye, intellectual disability reported as “low IQ”, “anger issues”, and possible schizophrenia. However, no medical records are available for review as the mother is estranged from the adoptive families of the siblings. Attempts to contact the biological mother through the adoptive families were unsuccessful. She is also reported to have prior poly‐drug use and to have been incarcerated. Ethnicity is reported to be Native American (Choctaw) and Caucasian, non‐Hispanic. Paternal history is unknown for all but one of the half‐siblings.

### Conventional and molecular cytogenetics

Microarray was originally ordered by independent referring physicians on Sibling‐8, Sibling‐9, and Sibling‐10, due to learning delay and speech delay. Communication with the ordering physicians confirmed that the patients originated from a large half‐sibling cohort that had been either adopted or in foster care. Referral for genetic counseling was recommended to discuss the microarray analysis result with the adoptive and foster families. Family history obtained at genetic counseling confirmed that the three patients were maternal half‐siblings and that they had eight additional half‐siblings. Fluorescence in situ hybridization (FISH) analysis was performed (using BAC clone probe RP11‐29N16) on the sample of the initial proband (Sibling‐8) to confirm that FISH for 4p16.1 (BAC clone RP11‐29N16) was informative in this family. This confirmed the deletion and facilitated testing in other siblings. Subsequent testing was performed either by FISH analysis for 4p16.1 (BAC clone RP11‐29N16) or microarray, depending on the preference of the ordering provider. Because Sibling‐4 previously had microarray analysis with a normal result in 2009 (Nimblegen 385K chip, genome assembly Mar 2006 [NCBI 36/Hg18]), FISH analysis was ordered and was negative for the familial deletion.

## Results

### Microarray analysis

Human reference genomic DNA was obtained through Agilent Technologies (Agilent Technologies, Santa Clara, CA, USA). The patients’ DNA and reference DNA were labeled with either cyanine 3 (Cy‐3) or cyanine 5 (Cy‐5) following the standard protocol provided by Agilent. Equivalent labeling DNA products were mixed together and were loaded onto Agilent's 2x400K oligo microarray chip which is built based on GRCh37/hg19 (Agilent Technologies, Santa Clara, CA, USA). The slide was incubated in a hybridization oven at 67°C for 40 h. Slides were then washed and scanned using a NimbleGen MS 200 Microarray Scanner (NimbleGen System Inc, Madison, WI, USA). The image was analyzed using CytoGenomics 2.7 software (Agilent Technologies, Santa Clara, CA, USA). Significant gains or losses reported were those in which included genomic copy number losses of >200 kb and gains of >500 kb outside a known clinically significant region, and that included at least one OMIM (http://www.ncbi.nlm.nih.gov/omim) annotated gene.

Microarray analysis was performed on venous blood samples from four of the siblings. The familial 4p16.1 deletion was identified in three of the siblings by microarray analysis (Data S1). A fifth sibling (Sibling‐4) had microarray analysis performed in 2009 and FISH analysis was performed to confirm the normal result.

### FISH analysis

Fluorescence in situ hybridization (FISH) analysis was performed following standard clinical laboratory protocol on venous blood samples from five of the siblings. FISH for 4p16.1 deletion (BAC clone RP11‐29N16) was first performed on the proband (Sibling‐8). Once the results were confirmed as informative, FISH analysis for the familial 4p16.1 deletion was performed on four of the half‐siblings and identified the familial deletion in three of the five tested (Data S2).

### Genotype–phenotype correlation

Clinical features of each sibling were compared and contrasted according to presence or absence of the familial 4p16.1 deletion (Table 1). Clinical features were also compared to those known to be associated with Wolfram‐like syndrome (OMIM #614296) (Table 1). Clinical history revealed several consistent features among those with the deletion, including astigmatism, gross and fine motor delay, variable intellectual disability, and variable psychological concerns. However, two of the siblings who were not found to have the deletion also had learning delays and various psychological concerns. Thus, variable phenotypic expression was seen in this family. No clear pattern of features associated with Wolfram‐like syndrome was found. Thus, the clinical significance of deletion of 4p16.1 remains unclear.

## Discussion

We present a large, maternal half‐sibling cohort with a familial deletion of 4p16.1. Eleven maternal half‐siblings were identified following independent microarray analysis in three of the siblings, followed by confirmation of FISH analysis (BAC clone RP11‐29N16) in the proband. Seven of the siblings were consented to the IRB approved Chromosomal Anomalies Registry, four of the siblings were identified as having the familial deletion, and three were negative for the deletion. Review of clinical features revealed no clear pattern of features associated with the deletion and no similarity to those known to be associated with Wolfram‐like syndrome.

This study represents the first clinical report of a large, sibling cohort involving deletion of 4p16.1. Review of published literature revealed no prior clinical reports of a similar deletion. The deleted region in this report includes the gene *WFS1* (OMIM #606201) which is associated with Wolfram syndrome (OMIM #222300) 1. However, the siblings’ reported clinical features are not consistent with Wolfram syndrome, nor do they fit the autosomal dominant Wolfram‐like syndrome (OMIM #614296).

Though unlikely, given the reported family structure, we are not able to rule out the possibility of an underlying sequence mutation not detectable by microarray. Additional testing such as targeted panel or whole exome sequencing could be considered in this family to rule out such an underlying genetic alteration contributing to the phenotype described in this cohort. However, due to family circumstances and coverage of such testing, this was not pursued at the time of this report.

The clinical significance of deletion of 4p16.1 remains unclear, both in the published literature and in the family reported here. Patients with similar deletions may have variable phenotypic expression, even with identical copy number changes. To date, this is the first clinical report of a large sibling cohort with deletion of 4p16.1 detected by microarray analysis. We present this report to aid in the characterization of the clinical significance of interstitial deletions on the short‐arm of chromosome 4 in this family and in others.

## Conflict of Interest

None declared.

## Supporting information


**Data S1.** Microarray analysis revealing 405KB deletion of 4p16.1.

**Data S2.** FISH analysis revealing deletion of 4p16.1.
