# Supplementary figures and images for: Clinical report: variable phenotypic expression in a large sibling cohort with a deletion of 4p16.1
Source: Clin Case Rep. 2016 Aug 18;4(10):913–8. doi: 10.1002/ccr3.638 (PMC5054462; doi:10.1002/ccr3.638)

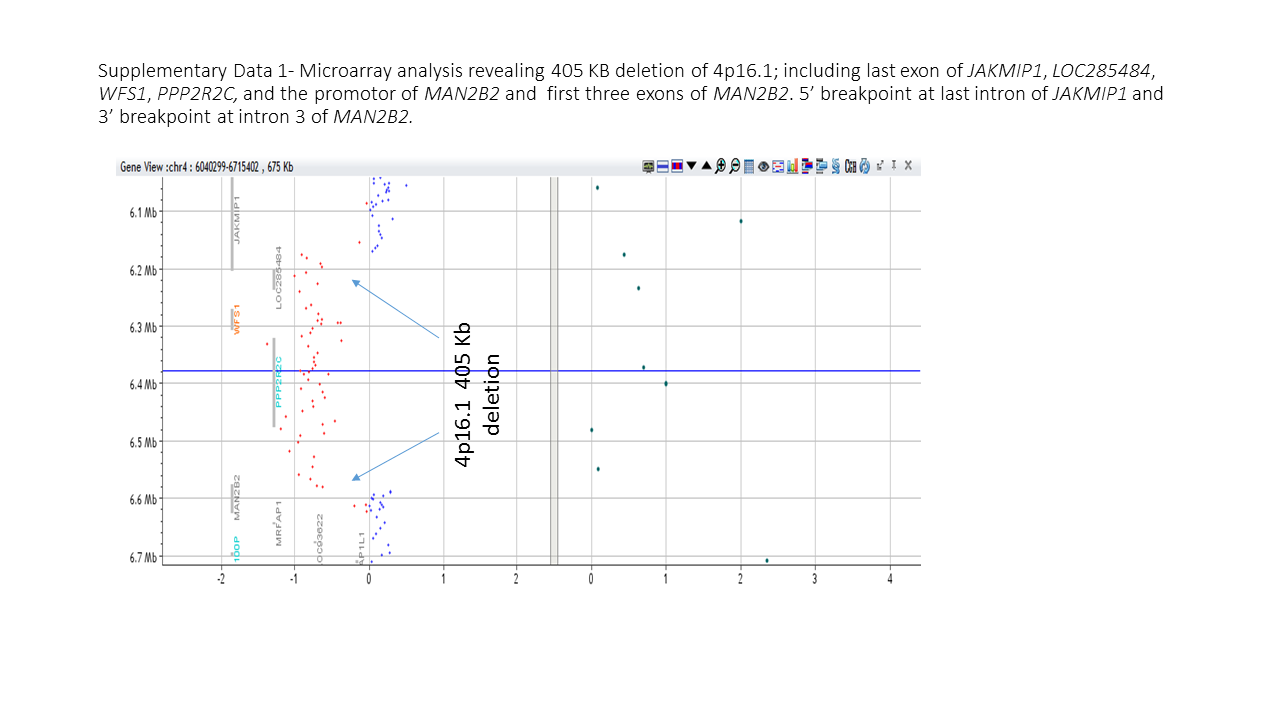

Supplement: Supplementary file 1 — Data S1. Microarray analysis revealing 405KB deletion of 4p16.1. [file CCR3-4-0913-s001.tif]

Supplementary Data 2- FISH analysis revealing deletion of 4p16.1


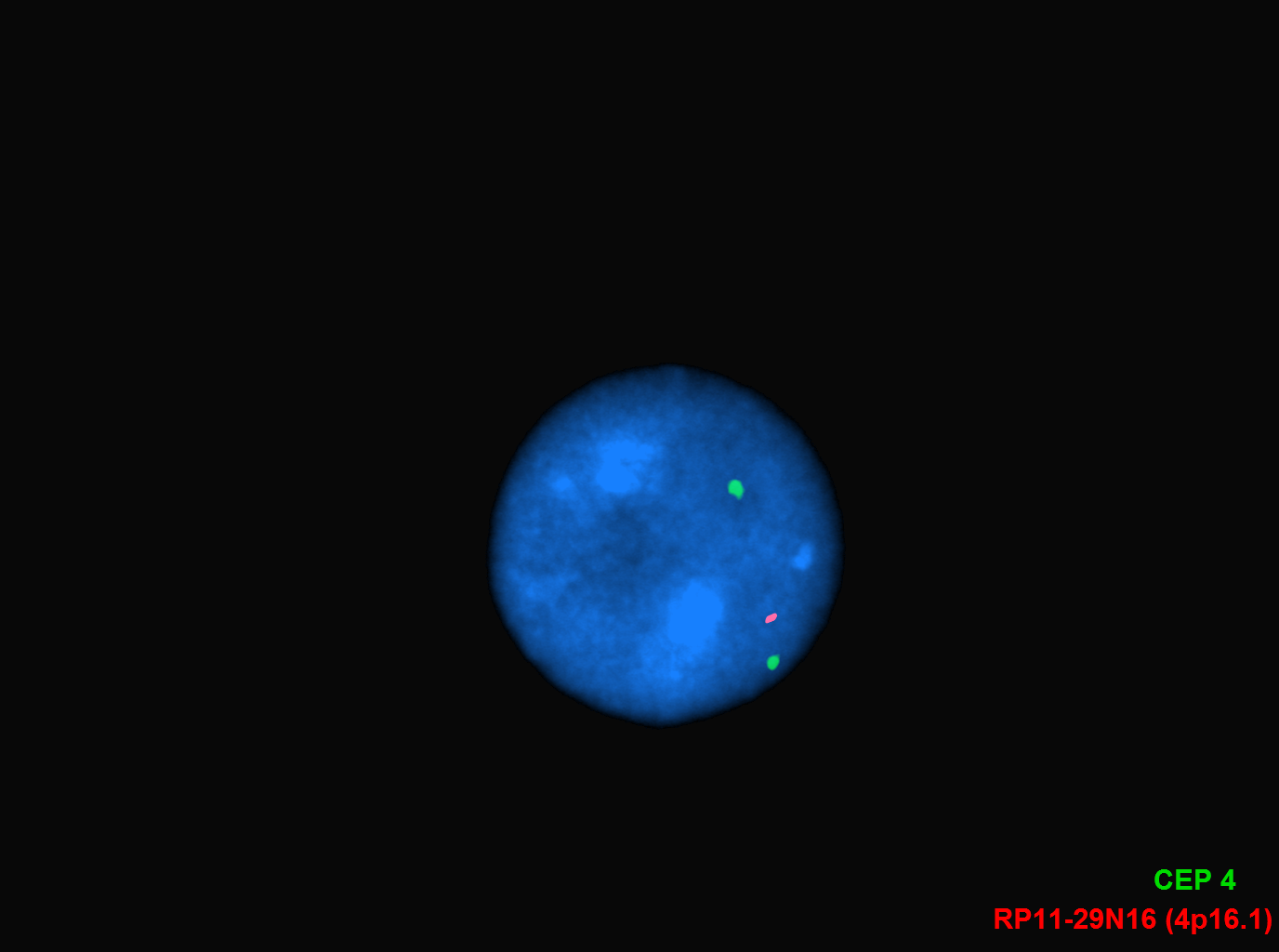

Supplement: Supplementary file 2 — Data S2. FISH analysis revealing deletion of 4p16.1. [file CCR3-4-0913-s002.docx]
